# Alternating current electrical stimulation enhanced chemotherapy: a novel strategy to bypass multidrug resistance in tumor cells

**DOI:** 10.1186/1471-2407-6-72

**Published:** 2006-03-17

**Authors:** Damir Janigro, Catalin Perju, Vincent Fazio, Kerri Hallene, Gabriele Dini, Mukesh K Agarwal, Luca Cucullo

**Affiliations:** 1 Division of Cerebrovascular Research, Cleveland Clinic Lerner College of Medicine, Cleveland, OH 44106 –, USA; 2 Department of Molecular Genetics, Cleveland Clinic Lerner College of Medicine, Cleveland, OH 44106 –, USA; 3 Department of Neurosurgery, Cleveland Clinic Lerner College of Medicine, Cleveland, OH 44106 –, USA; 4 Department of Molecular Medicine, Cleveland Clinic Lerner College of Medicine, Cleveland, OH 44106 –, USA

## Abstract

**Background:**

Tumor burden can be pharmacologically controlled by inhibiting cell division and by direct, specific toxicity to the cancerous tissue. Unfortunately, tumors often develop intrinsic pharmacoresistance mediated by specialized drug extrusion mechanisms such as P-glycoprotein. As a consequence, malignant cells may become insensitive to various anti-cancer drugs. Recent studies have shown that low intensity very low frequency electrical stimulation by alternating current (AC) reduces the proliferation of different tumor cell lines by a mechanism affecting potassium channels while at intermediate frequencies interfere with cytoskeletal mechanisms of cell division. The aim of the present study is to test the hypothesis that permeability of several MDR1 over-expressing tumor cell lines to the chemotherapic agent doxorubicin is enhanced by low frequency, low intensity AC stimulation.

**Methods:**

We grew human and rodent cells (C6, HT-1080, H-1299, SKOV-3 and PC-3) which over-expressed MDR1 in 24-well Petri dishes equipped with an array of stainless steel electrodes connected to a computer via a programmable I/O board. We used a dedicated program to generate and monitor the electrical stimulation protocol. Parallel cultures were exposed for 3 hours to increasing concentrations (1, 2, 4, and 8 μM) of doxorubicin following stimulation to 50 Hz AC (7.5 μA) or MDR1 inhibitor XR9576. Cell viability was assessed by determination of adenylate kinase (AK) release. The relationship between MDR1 expression and the intracellular accumulation of doxorubicin as well as the cellular distribution of MDR1 was investigated by computerized image analysis immunohistochemistry and Western blot techniques.

**Results:**

By the use of a variety of tumor cell lines, we show that low frequency, low intensity AC stimulation enhances chemotherapeutic efficacy. This effect was due to an altered expression of intrinsic cellular drug resistance mechanisms. Immunohistochemical, Western blot and fluorescence analysis revealed that AC not only decreases MDR1 expression but also changes its cellular distribution from the plasma membrane to the cytosol. These effects synergistically contributed to the loss of drug extrusion ability and increased chemo-sensitivity.

**Conclusion:**

In the present study, we demonstrate that low frequency, low intensity alternating current electrical stimulation drastically enhances chemotherapeutic efficacy in MDR1 drug resistant malignant tumors. This effect is due to an altered expression of intrinsic cellular drug resistance mechanisms. Our data strongly support a potential clinical application of electrical stimulation to enhance the efficacy of currently available chemotherapeutic protocols.

## Background

Multidrug resistance is often the result of overexpression of membrane glycoproteins known as P-glycoprotein (P-gp) [1]. In humans, the drug transporter P-gp is encoded by the MDR1 gene while two encoding genes Mdr1a and Mdr1b, [2] are present in rodents. P-gp proteins belong to the super family of ATP-binding cassette transporters [3]. MDR proteins recognize a wide range of substrates of diverse chemical structure. This lack of substrate specificity explains the cross-resistance to several chemotherapeutic compounds, the characteristic clinical feature found in a multi-drug resistance phenotype. In addition to their overlapping substrate specificity, each transporter can handle a variety of unique compounds.

Pgp-MDR1 has been identified as an obstacle to both peripheral and CNS chemotherapy [4,5]. Thus, drug refractory tumors present a clinical challenge which requires novel treatment modalities [6,7]. In fact, despite recent advances in chemotherapy, the true potential of new agents has not been realised due to the multitude of resistance pathways that impair chemotherapeutic efficacy. Taken together, these findings demonstrate that: 1) MDR is often an insurmountable impediment to cancer chemotherapy; 2) In the case of brain tumors, MDR1 acts by a dual mechanism: a pharmacokinetic one mediated by endothelial cells at the blood-brain barrier and a pharmacodynamic one at the tumor cell plasma membrane where multidrug resistant transporters are widely expressed.

Limitations of current therapies are well known and still far from being addressed. For example, radiotherapy produces its biologic effect on cancerous tissue by ionization. Radiation therapy is carried out by megavolt energy radiation in the form of X-rays from a linear accelerator or gamma rays from a cobalt source. Radiation is highly penetrating, but in order to reach the region of the tumor being treated, the radiation beam must pass through regions containing healthy tissue and may therefore destroy these as well as the malignant tumor. Chemotherapy is limited by drug resistance [1,7,8] and by the fact that malignant and non-malignant cells are so similar that it is difficult to destroy cancerous cells without concurrently destroying healthy cells. The adverse effects of chemotherapy are notorious. Increasing the temperature of the tumor to a level at which cancerous cells are destroyed can be used to destroy malignant tumors. One method used for this purpose is to focus a beam of microwave energy, of the type generated in a microwave oven, onto the tumor. The drawback of this technique is that healthy tissues through which the beam must pass to reach the tumor have higher moisture content than the interior of the tumor and are therefore more reactive to microwave energy. Surgical approaches to excise a malignant tumor are limited by location of the tumor, as in the case of tumors in the brain, which are often inoperable. But even where the tumor is accessible, residual malignant cells are left behind, leading to recurrence.

Other approaches include the electrical stimulation in or around the tumor [9,10], biological response monitors, or specific MDR1 inhibitors such us Tariquidar (XR9576) [11]. In an electrochemical procedure [10,12], electrodes are implanted in or around the malignant tumor to be treated. The treatment lasts for several hours during one or more sessions and can be used either alone or in conjunction with other therapy such as chemotherapy or radiation therapy. Applied across these electrodes is a low DC voltage of about 6 ± 10 V with currents of 40 ± 100 mA, causing a current to flow between the platinum electrodes through the tumor. The exposure of living tissue to this electric field leads to a wide range of effects such as: 1) Water migrates from the anode to the cathode; 2) The "anodic site" in the tissue becomes strongly acidic and the cathodic site strongly alkaline; 3) Protein denaturation; 4) Bleaching of the local tissue and 5) Cell metabolism and its existing environment are disturbed severely by the electrochemical treatment, causing the destruction of both normal and tumor cells rapidly and completely [10,12,13].

More recently [see Additional file 1], very low-intensity (< 15 μA), low frequency AC (50 HZ) applied to dividing cells was shown to inhibit the cell cycle by affecting potassium channels [14] while another study has revealed that at low-intensity, intermediate-frequency (100 – 300 kHz) AC electrical fields may interfere with cytoskeletal mechanisms responsible for the formation of mitotic spindles [15], thus preventing tumor proliferation. In this context however, a cytostatic effect is not sufficient to decrease tumor burden if chemotherapy is ineffective. We now describe a new approach based on delivery of low frequency, low intensity AC stimuli directly to the tumor cells to achieve increased sensitivity to common chemotherapic agents in spite of robust MDR1 expression.

## Methods

### Cell culture and electrical stimulation set up

Rat glioma C6 (Cat# CCL-107), prostate tumor (PC-3, Cat# CRL-1435), lung tumor (H1299 Cat# CRL-5803), fibrosarcoma (H1080 Cat# CRL-12012) and ovarian cancer (SKOV-3 Cat# HTB-77) cell lines were purchased from ATCC and characterized for MDR1 expression. Cells were initially expanded in Dulbecco's modified essential medium (DMEM-F12) supplemented with 2 mM glutamine, 10% fetal bovine serum (FBS), 100 units of penicillin G sodium per ml, and 100 μg of streptomycin sulfate per ml. All cells were maintained at 37°C in a humidified atmosphere consisting of 5% CO_2 _and 95% air. The cells were seeded into pre-coated (3 μg/cm^2 ^Poly-d-Lysine) 24 well plates with average seeding density being 1 × 10^4 ^cells/cm^2 ^in every experiment. Well plates were engineered to accommodate stainless steel electrodes, which were connected to a computer controlled waveform generator via a programmable I/O board. We used a dedicated program to generate and monitor the electrical stimulation protocol. Cultures were stimulated for 3 days after the initial cell seeding. Media samples were taken on a daily basis and processed for AK measurements. Cellular growth was monitored every day by inspection with phase contrast microscopy.

### Choice of the electrical stimulation paradigms

Cells undergoing stimulation protocols were exposed to 50 Hz AC (7.5 μA 32 cycles/pulse, 10 sec interval between pulses) for three days. The stimulation paradigms presented herein (frequency, intensity interstimulus interval) originate from a previous study [see Additional file 1] aimed at demonstrating the cytostatic effect promoted by the exposure to AC current of tumor cell lines. We shown that cells exposed to AC stimulation at 10 Hz, ≈ 10 μA for 2 to 5 days grew at a rate similar to non-stimulated controls. In contrast, stimulation at 25 – 100 Hz caused a pronounced decrease in cell proliferation as early as three days after stimulation. The effects persisted and amplified with prolonged exposure to electric pulses. However, while stimulation up to 50 Hz decreased cell number through a direct effect on cell cycle, at frequencies greater than 50 Hz and or intensities greater than 15 μA the effect on cell proliferation significantly overlapped with a cytotoxic one on both tumors and normal cells. In view of a possible clinical application of this novel approach, limitation of peripheral "normal" cells damage due to a possible exposure to the electric field was vital to our project and was as well an element of distinction from the classical electrochemical protocol.

### Cell isolation, characterization and primary culture

MDR1 over-expressing astrocyte cultures were established from human cerebral cortical tissue of patients undergoing temporal lobectomies to relieve medically intractable seizures. Brain resections were collected in an ice-cold artificial cerebrospinal fluid solution bubbled with 5% CO_2_and 95% O_2_. This solution consisted of (in mM): 120 NaCl, 3.1 KCl, 3 MgCl_2_, 1 CaCl_2_, 1.25 KH_2_PO_4_, 26 NaHCO_3_, 10 dextrose. Briefly, tissue was homogenized for 20 min. at 37°C after gentle trituration and incubation in phosphate buffer saline (PBS) containing trypsin (0.2%)/DNase (1 mg/ml, Sigma-Aldrich, MO, USA). After centrifugation (200 g for 5 min.) and filtration through 70 μm nylon sieve, cells were seeded in appropriated poly-D-lysine coated flask. The culture medium consisted of Dulbecco's modified essential medium (DMEM) supplemented with 10% FBS and 2 mM glutamine, 100 U/ml penicillin G sodium and 100 μg/ml streptomycin sulfate. Immunological characterization was performed with rabbit polyclonal antibodies that recognize the glial marker GFAP (Dako Corporation, Carpentaria, CA, USA) and with human anti-P-Glycoprotein polyclonal antibody (1:100, Calbiochem-Novabiochem Corporation, San Diego, CA, USA) to assess for MDR1 expression. Normal astrocytes from ScienCell (Cat# 1810), were used as control (Normal Astro).

### Current density calculation

The peak surface current density was calculated by an automated 2-D finite-elements approximation of the system. In the frequency range of interest, the system can be considered purely resistive due to the lack of inductive or capacitive effects, thus allowing the use of a simplified model. Each well or Petri dish was divided into 360 sub-elements, and Kirchhoff's laws applied according to: ∑I = 0 (for every node) and ∑V = 0 (for every closed loop). The resulting current, divided by the element area, allowed the calculation of the surface current density.

### Adenylate Kinase measurement

Detection of cytotoxicity and cytolysis was assessed by measurement of AK release. Media samples were taken before and after the experiment. The measurements were performed by the use of the ToxiLight™ HS kit (Cambrex Bio Science Rockland, Inc.). The assays were conducted at ambient temperature (18–22°C) following the procedure described by the manufacturer. In brief, the assay method involves the release the AK into the surrounding matrix whenever cell damage occurs and the integrity of the plasma membrane is compromised. The AK enzyme following the introduction of an excess of ADP then generates ATP. Luciferase/luciferin is added to the sample, light is emitted in the presence of the ATP and the photon emission is measured using a luminometer.

### MDR1 immunohistochemical detection and distribution

To investigate the expression of MDR1 protein and its localization, cells were cultured on poly-d-lysine pre-coated slides. The slides were positioned in the electrified well plate and stimulated for 3 days. Cells were fixed in 4% formaldehyde at room temperature for 20 minutes and then washed three times with 1× PBS. Blocking was performed at room temperature for 1 h with: 0.3% Triton-X, 3% bovine serum albumin, 3% normal goat serum and 1× TBS. The primary antibody used was Anti-P-Glycoprotein (C494) hamster and human (mouse) monoclonal (1:40; Calbiochem, San Diego, California). The secondary antibody used was Fluorescein (FITC)-conjugated affinipure donkey anti-mouse IgG (1:200; Jackson Immunoresearch Laboratories, West Grove, Pennsylvania). Cellular distribution of MDR1 was assessed with the use of a 35-mm camera mounted on a fluorescent microscope unit (Leica Leitz DM-RXE) and interfaced to a PC (using Qcapture software – Quantitative Imaging Company). The images were analyzed by Phoretix 2D Image Analysis Software. Data were further analyzed using Origin Lab 7 software. Experiments were performed in triplicate.

### Doxorubicin uptake measurement, toxicity and cell viability

Parallel 24 well plate cultures (stimulated; pre-treated with the MDR1 blocker XR9576 [20 nM] and a control) were used in triplicates per each tumor cell type. Cells were exposed to different concentrations (1, 2, 4 and 8 μM) of doxorubicin for 3 hours. Media samples were collected before and after the exposure to doxorubicin to assess for AK release. Media containing doxorubicin was then washed out and replaced with cold media and kept at 4°C in order to inhibit MDR1 activity [16,17]. Cells were then exposed to Calcein (Calcein AM Fluorogenic Esterase Substrate Cell Viability and Cytotoxicity kit -Invitrogen, Carlsbad – CA, USA) according to the manufacturer protocols and specifications to assess for cell viability. In the absence of efflux activity, free calcein accumulates within the cell resulting in a 100- to 500-fold increase in the intracellular concentration of the dye and bright fluorescence exhibited by living target cells. Cell viability and quantification of the intracellular levels of doxorubicin were assessed by measurement of fluorescence intensity and compared to parallel XR9576-treated cultures and controls. Data were analyzed by Phoretix 2D Image Analysis Software and Origin Lab 7. The final experiment with normal and MDR1 over-expressing astrocytes was performed by increasing the concentration of XR9576 to 1 μM.

### Isolation of cellular fractions

Extraction of proteins from stimulated and control cell cultures were performed by the use of ProteoExtract Subcellular Proteome Extraction Kit (EMD Biosciences San Diego, CA Cat# 539790) designed for fast and reproducible extraction of subcellular proteomes from mammalian tissue and adherent and suspension-grown cells. In the specific case of adherent cells, the procedure is performed directly in the tissue culture dish without the need for cell removal. Cells or the parts of the cells remain attached to the plate during sequential extraction of subcellular compartments until the appropriate extraction reagent is used. Thus, the early destruction of the cellular structure by enzymatic or mechanical detachment of cells from the tissue culture plate and any mixing of different subcellular compartments is prevented. The assay was performed following the procedure described by the manufacturer. The stepwise extraction delivers four distinct protein fractions from one sample: 1) Cytosolic protein fraction; 2) Membrane/organelle protein fraction; 3) Nuclear protein fraction; 4) Cytoskeletal protein fraction. Proteins were obtained in the native state and processed for Western blot and protein analysis.

### Western Blot Analysis

Proteins differentially extracted from stimulated and control cells were re-dissolved in RIPA buffer containing protease inhibitors (0.17 mg/mL PMSF, 2 μg/mL leupeptin, and 0.7 μg/mL aprotinin). Prior to electrophoresis, protein extracts were denatured by heating at 100°C for 5 minutes in a running buffer solution containing RIPA, β-mercaptoethanol, and bromophenol blue tracking dye. 15 μg proteins were loaded in each lane. Duplicate acrylamide gels (12%, precast gels; Bio-Rad Labs, Hercules, CA) were run for 2.5–3 hrs at constant voltage (80 V) until the bromophenol blue tracking dye migrated to the bottom edge of the gels. Proteins were then transferred onto a blot of PVDF using constant current (40 mA) overnight at 4°C. Proteins were probed overnight at 4°C with primary MDR1 mouse anti-human antibody (1:100; Calbiochem Clone C494, San Diego, CA). Blots were washed and treated with rabbit anti-mouse IgG HRP conjugated secondary antibody (1:5000; Dako Corp., Carpinteria, CA). To ensure that the same amount of total protein was electroblotted, PVDF membranes were incubated for 20 minutes at 37°C in a "stripping buffer" (Restore Western Blot Stripping Buffer, Pierce, Rockford, IL). Non-specific binding blocking was performed as described above; membranes were reprobed with monoclonal anti β-Actin antibody (1:10,000, Clone AC-15, Sigma-Aldrich, St. Louis, MO). Protein bands were analyzed by Phoretix 2D Image Analysis Software and Origin Lab 7.

## Results and discussion

**Figure 1:**
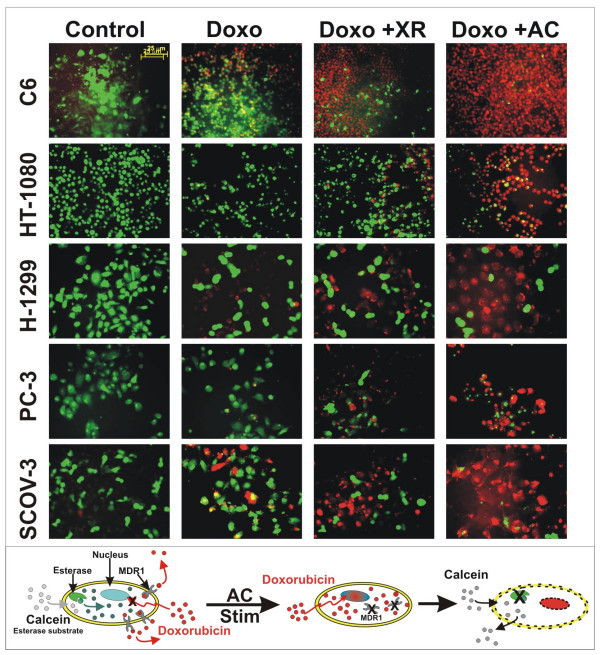
**Blockade of MDR1 by either XR9576 or electrical stimulation increases doxorubicin uptake. **Calcein stains (green fluorescence) cells that retain intact membrane structure, but it does not label dead cells and is rapidly lost under conditions that cause cell lyses. Doxorubicin (in red) is normally extruded by drug resistant mechanisms and thus enters untreated cells reluctantly. Note that exposure to either MDR blocker or electrical stimulation achieved a greater uptake of red fluorescent doxorubicin and dramatically decreased the number of viable cells (in green). **Small diagram**: In viable cells, hydrolysis of Calcein-AM by intracellular esterases produces fluorescent calcein that is retained in the cell cytoplasm. Exposure to AC stimulation increases doxorubicin cytotoxicity thus compromising cell viability and esterases activity.

**Figure 2:**
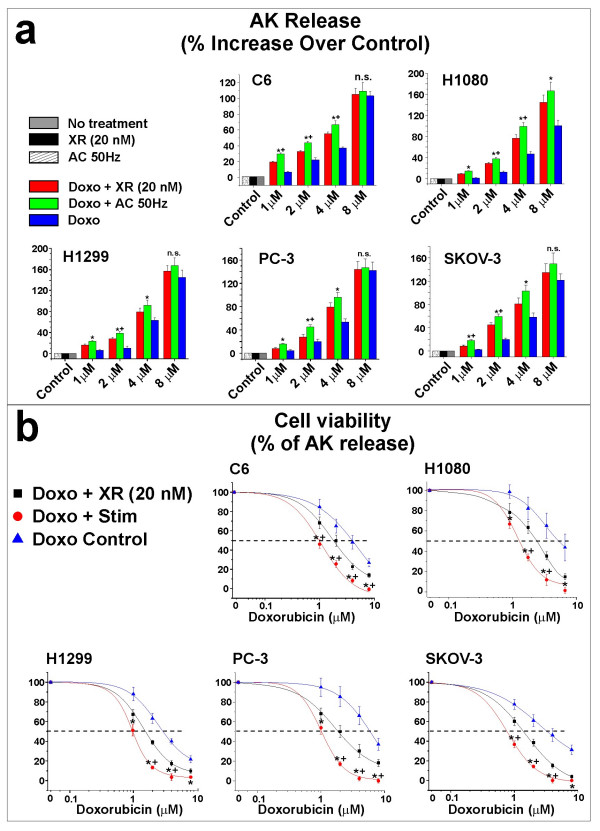
**AC stimulation enhance doxorubicin efficacy. (a) **Determination of doxorubicin toxicity by AK. As doxorubicin concentration increased, cells underwent significant detrimental changes. These were further increased by either MDR1 blockade or electrical stimulation. The asterisk refers to a statistical significant difference (n = 10; p = 0.028 by ANOVA) with non-stimulated cultures while the positive sign refers to statically significant differences (n = 10; p = 0.036) with cultures exposed to XR9576. Note that AC stimulation or exposure to XR9576 does not cause any cytotoxic effect in comparison to non-treated (control) cultures. (**b**) Quantitative analysis of the experiment shown in figure (1), including adult response for the chemotherapeutic agent doxorubicin. Note that electrical stimulation achieved an equal or greater effect than blockade of MDR1 by XR9576. The asterisk indicates a significant difference (n = 10; p = 0.023 by ANOVA) from controls whereas the sign plus symbolizes significant difference (n = 10; p = 0.031) from XR-treated cultures. Data points were fitted by the following sigmoid: Equation: y = A2 + (A1-A2)/(1 + (x/x0)^)

Cells undergoing stimulation protocols were exposed to 50 Hz AC (7.5 μA 32 cycles/pulse, 10 sec interval between pulses) for three days as described in the method section. We have previously shown that this stimulation paradigm is devoid of temperature-dependent effects, is not cytotoxic and specifically does not disrupt cell membrane integrity [14]. After 72 hours of continuous stimulation, cells were exposed for 3 hours to increasing concentrations (1, 2, 4, and 8 μM) of doxorubicin, an MDR1 substrate and potent chemotherapeutic drug to which these cell lines are exquisitely resistant (e.g., HL-60 cells) [18]. Cell viability was assessed by determination of adenylate kinase (AK) release [19] and calcein uptake [20]. Since calcein is a well known MDR1 substrate, this latter experiment was carried out at 4°C in order to inhibit MDR1 activity [17], allow for intracellular accumulation of calcein and assess for cell viability as described in the method section. The results shown in Figure 1a demonstrate that AC stimulation increased doxorubicin uptake (in red) leading to increased cell damage even at low concentrations as demonstrated by AK release measurements (Figure 2a). The sensitivity of this method is improved due to the equilibrium reaction being artificially driven to overproduce ATP by the addition of an excess of ADP. Moreover, unlike traditional ATP assays, AK levels are constant and do not fluctuate with metabolic state, providing a more reproducible, quantifiable signal which closely correlates with cell numbers. These features enable small changes in the cell population to be detected.

The effects of AC stimulation on cell viability were compared to those of the MDR1 inhibitor XR9576 (20 nM) [11,21] concomitantly applied to doxorubicin. We found that AC stimulation affected cell survival more potently than pre-treatment with the MDR1 inhibitor. This was demonstrated by the analysis of cell cultures exposed to fluorescent doxorubicin followed by calcein uptake at low temperature. The effect of AC stimulation on cell viability is particularly evident at low doses of doxorubicin (1 and 2 μM, Figure 2b), since the differences were less obvious at higher concentrations of doxorubicin. The fact that 20 nM concentrations of XR exhibited an effect on cell viability capable of greatly enhancing the toxicity of doxorubicin suggest two things: 1) The intrinsic resistance to doxorubicin was almost entirely overridden by the specific MDR1 blocker XR9576 thus ruling out a significant contribution of MRP and 2) That the electrical stimulation applied had an effect much greater than that of XR9576 applied at EC50 level. Taken together these considerations imply that had we used much higher concentration of XR9576 we would have achieved the same effect as with electrical stimulation.

**Figure 3:**
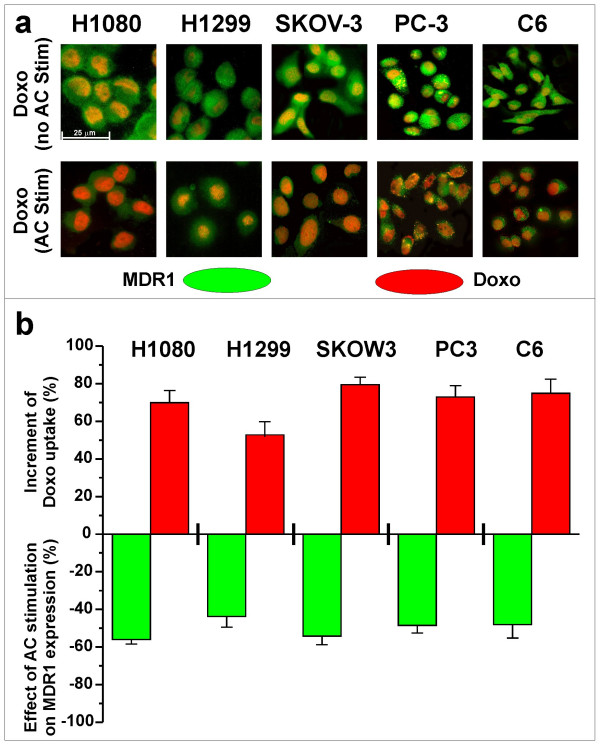
Inverse correlation between the expression of MDR1 and the intracellular uptake of doxorubicin. **(a) **Micro photographs showing the accumulation of doxorubicin (in red) in cells also stained for MDR1 immunoreactivity (in green). Note that electrical stimulation greatly increased the accumulation of doxorubicin in the cell nucleus while achieving a proportionate decrease of immunological reactive signal for MDR1. **(b) **Data summarizing the inverse relationship between MDR1 expression and doxorubicin uptake.

Immunohistochemical and fluorescence analysis of cells exposed to doxorubicin following AC stimulation revealed a significant decrease in MDR1 expression paralleled by an increased intracellular uptake of doxorubicin (Figure 3a). Figure 3b shows the relationship between MDR1 expression and the intracellular accumulation of doxorubicin. As expected in control cells the binding of Doxorubicin was nuclear, presumably involving DNA or other nuclear material. These bindings are not easily reversed, and therefore one expects persistence of the Doxorubicin signal even after manipulation of the cell membrane. We have used the same immunocytochemical approach to process cells prior and after stimulation. As can be clearly seen in figure 3a, following AC stimulation and under reduced MDR1 load increased Doxorubicin binding to the nuclear material was observed.

**Figure 4:**
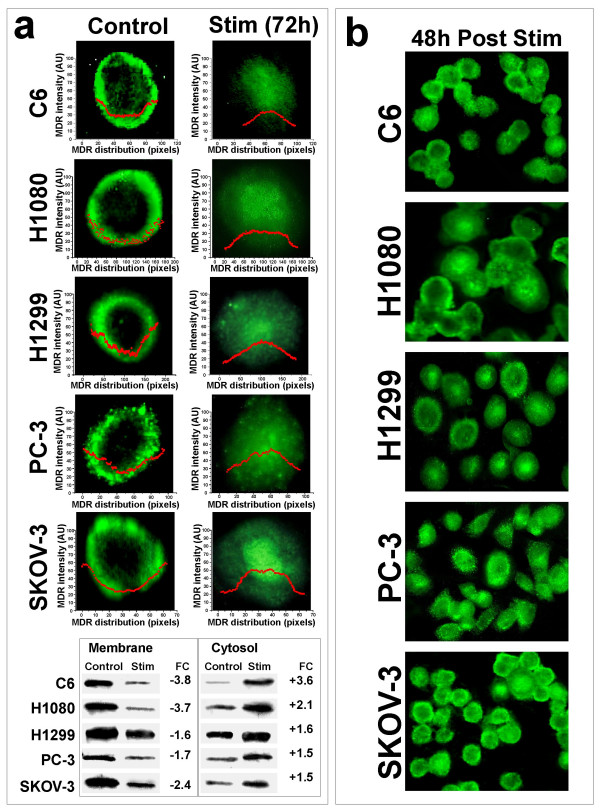
**Cellular MDR1 distribution is modulated by electrical stimulation. a) **The **upper panel **shows the immunocytochemical analysis of sub-cellular expression of MDR1. Note that prior to stimulation, MDR1 was primarily located in proximity of the plasma membrane. In stimulated cells, however, MDR immunosignal significantly shifted towards the center of the cell, suggesting a cytoplasmic or nuclear localization. Quantification was achieved by plotting the levels of expression against cell diameter; the dotted red lines show these relationships. Western blot analysis (**lower panel**) of sub-cellular fractions of cell types used for the experiments. Note that electrical stimulation caused a dramatic shift of membrane-bound MDR1 towards the cytosolic compartment. The numbers to the right show fold changes after electrical stimulation. **b) **Immunocytochemical analysis of MDR1 sub-cellular expression 48 hours post stimulation. Note that the effect of AC stimulation on MDR1 is reversible. Cells previously exposed to AC field demonstrating MDR1 cytosolic accumulation now replicates generating "normal" MDR1 + cells.

When the cellular distribution of MDR1 was investigated by computerized image analysis and immunohistochemistry, we discovered that AC stimulation not only decreased the expression of MDR1, but also altered its sub-cellular distribution. In non-stimulated cultures, MDR1 was found to be primarily expressed close to the plasma membrane [22], while in stimulated cultures we observed MDR1 expression predominantly in the cytosolic compartment (Figure 4a). The change in MDR1 distribution was indeed confirmed by Western blot analysis performed on membrane and cytosolic protein fractions (Figure 4a lower panel). This shift toward the cytosolic compartment may explain the loss of drug extrusion potency.

While one is tempted to presume that what happened is translocation from the membrane to the cytosol, our current hypothesis is that electrical stimulation achieved the opposite effect, for example impeding release of MDR1 from the cytosolic protein synthesis machinery and thus preventing correct insertion in the membrane. This seems to be so far the more appropriate explanation since the effects of electrical stimulation become evident only after several proliferative cycles (approximately 2 days). Furthermore, as shown in figure 4b the effects of AC stimulation are reversible. Cell cultures that have been exposed to the AC protocol for 3 day and then allowed to rest for 48 h demonstrate a mixed population of cells where the functional MDR1 distribution appears to be partially restored. It is also to be noted that AC stimulation concurrently decreases cell proliferation as demonstrated in our previous work [14]. This explains the apparently long exposure time to AC electric field required for the stimulation to achieve an effect (3 days) and the relatively long time for the cells to recover.

**Figure 5:**
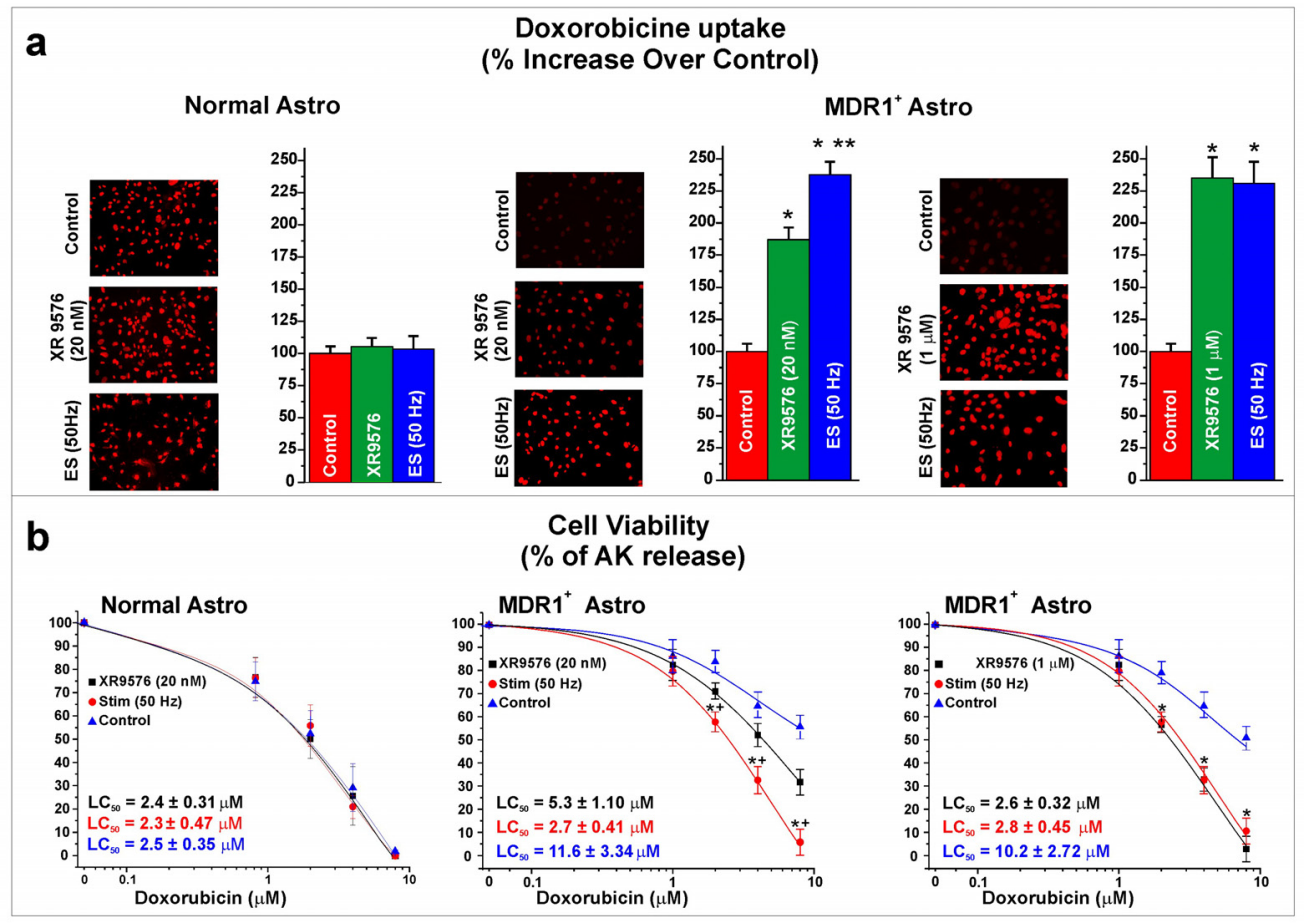
**Electrical stimulation selectively increased chemotherapeutic efficacy in drug resistant cells**. **(a) **Doxorubicin uptake in normal human astrocytes versus MDR1 over-expressing glial cells isolated from patients with multiple drug resistant epilepsy. Note that electrical stimulation and MDR1 blockade by XR9576 (20 nM and 1 μM) significantly increased doxorubicin uptake in MDR1^+ ^cells compared to controls while normal astrocytes were virtually unaffected as confirmed by quantitative analysis (n = 10; p = 0.017). Note that the effect of AC stimulation on Doxorubicin uptake is comparable to the effect of XR used at a final concentration of 1 μM where full MDR1 blockade was achieved. **(b) **Estimation of lethal concentration of doxorubicin under different experimental conditions. Normal astrocytes were virtually insensitive to electrical stimulation and MDR1 blockade by XR9576 at all concentrations of doxorubicin tested. In contrast, MDR1 expressing glial cells responded to ES or MDR1 blockade. Note that a concentration of 20 nM of XR9576 corresponds to EC50. The final concentration of XR used for the comparison (1 μM) was approximately three times higher that the concentration required to achieve full blockade of the MDR1 transporters. The asterisk indicates a significant difference (n = 10; p < 0.022 by ANOVA) from controls whereas the sign plus symbolizes significant difference (n = 10; p < 0.027) from XR-treated cultures. Data points were fitted by the following sigmoid: Equation: y = A2 + (A1-A2)/(1 + (x/x0)^).

Ideally, a chemotherapeutic approach achieves high levels of target toxicity while sparing normal, healthy cells. In order to evaluate the specificity of AC for drug resistant cells, human brain astrocytes (non-MDR1 over-expressing) and drug-resistant, MDR1 over-expressing astrocytes isolated from drug refractory epileptic patients were exposed to doxorubicin (1 μM) for 3 hours following stimulation or pre-treatment with XR9576 at EC50 (20 nM) and over-saturating levels (1 μM) [21]. No significant differences of drug uptake were detected in normal astrocytes undergoing AC stimulation in comparison to control or XR9576-treated cultures (Figures 5a). However, MDR1 over-expressing astrocytic cultures exposed to the same experimental paradigms showed drug uptake patterns similar to that observed in tumor cell cultures. These data demonstrate that low frequency, low intensity AC stimulation is only effective when drug uptake is limited by MDR1 (Figure 5b).

In summary, we have shown that in addition to a cytostatic effect, AC stimulation also causes the accumulation of MDR1 protein into the cytosol (a non-functional placement) in disfavour of their functional localization on the plasma membrane. In addition, overall MDR1 expression was decreased in stimulated cells. These effects increased the cellular uptake of MDR1 drug substrates and ultimately boosted chemotherapic efficacy. The molecular mechanisms of this effect are yet unknown. There are several possible explanations including the potential involvement of a cytoskeletal mechanism in intracellular protein trafficking. The possibility that electrical stimulation inhibits the correct positioning of MDR1 in the cell membrane is supported by findings by others showing that electrical stimulation can affect microtubular structural assembly and spindle formation [15]. Based on this hypothesis, it is possible that MDR1 remains bound to either ribosomal proteins or other cytoplasmic compartments. Another significant finding of the study is the specificity of AC stimulation for cells expressing MDR1. Electrical stimulation did not increase the sensitivity of normal (non-drug resistant) cells to doxorubicin thus excluding the possibility that AC affects plasma membrane integrity. Furthermore, since several proliferative cycles are required in order for AC to be effective, cells which cell cycle is slow or almost inexistent (e.g. neurons) in comparison to tumors are practically unaffected by the exposure to the electric field. In addition to that, the effect of AC stimulation on the intrinsic drug resistance is reversible as is its anti-proliferative effect.

## Conclusion

The main finding presented here is that very low intensity AC current delivered in the proximity of malignant tumors can reduce drug resistance by a mechanism implicating impaired translocation of MDR1 to its functional localization at the plasma membrane. In addition, as previously demonstrated [14] the same stimulation protocol promotes a cytostatic effect reducing tumor cells proliferation. These findings suggest a potential application of low intensity AC current in the treatment of tumor growth by synergistically reducing neoplastic cell division and intrinsic tumor drug resistance. In view of the widespread use of stimulators and stimulating electrodes for the treatment of a variety of other diseases (such as treatment-resistant depression, epilepsy and Parkinson) [23-26], it seems possible that coupling electrical stimulation to current chemotherapy protocols will improve the efficacy of our therapeutic approach to neoplasms. AC stimulation may also decrease the quantity of chemotherapic drugs required to reduce tumor burden. This should reduce chronic exposure to chemotherapic agents as well as the side effects typically associated to chemotherapy. It is also to be noted that both the cytostatic effect and the reduced drug resistance are fully reversible.

## Competing interests

The author(s) declare that they have no competing interests.

## Authors' contributions

DJ co-supervised the project and participated in its design, coordination and data analysis. CP carried out the Western blot and cell culture experiments. VF carried out the Western blot and data analysis. KLH carried out the immunoassays. GD engineered the software and the hardware to perform the electrical stimulation experiment. MKA performed the cells characterization. LC conceived the study, elaborated its design, performed the computerized image acquisition data and the statistical analysis (with the collaboration of DJ). LC also performed most of the toxicity and doxorubicin uptake experiments and drafted the manuscript. All authors read and approved the final manuscript.

## Pre-publication history

The pre-publication history for this paper can be accessed here:



## Supplementary Material

Additional File 1 **Very low intensity alternating current decreases cell proliferation. **Electric fields impact cellular functions by activation of ion channels or by interfering with cell membrane integrity. Ion channels can regulate cell cycle and play a role in tumorigenesis. In the absence of thermal influences, low-frequency, low-intensity, alternating current (AC) directly affects cell proliferation without a significant deleterious contribution to cell survival. However, to be effective, exposure to AC stimulation also requires a permissive role for GIRK2 (or KIR3.2) potassium channels. Click here for file
