# Supplementary material for: Alternating current electrical stimulation enhanced chemotherapy: a novel strategy to bypass multidrug resistance in tumor cells
Source: BMC Cancer. 2006 Mar 17;6:72. doi: 10.1186/1471-2407-6-72 (PMC1435916; doi:10.1186/1471-2407-6-72)
Supplement: Additional File 1 — Very low intensity alternating current decreases cell proliferation. Electric fields impact cellular functions by activation of ion channels or by interfering with cell membrane integrity. Ion channels can regulate cell cycle and play a role in tumorigenesis. In the absence of thermal influences, low-frequency, low-intensity, alternating current (AC) directly affects cell proliferation without a significant deleterious contribution to cell survival. However, to be effective, exposure to AC stimulation also requires a permissive role for GIRK2 (or KIR3.2) potassium channels. [file 1471-2407-6-72-S1.pdf]

Very Low Intensity Alternating Current Decreases Cell Proliferation

LUCA CUCULLO,[1] GABRIELE DINI,[1] KERRI L. HALLENE,[1] VINCENT FAZIO,[1] ERIN V. ILKANICH,[1] CHIAZOR IGBOECHI,[1] KELLY M. KIGHT,[1] MUKESH K. AGARWAL,[2] MARY GARRITY-MOSES,[3] AND DAMIR JANIGRO[1,3,4*]

[1]*Division of Cerebrovascular Research, Cleveland Clinic Lerner College of Medicine, Cleveland, Ohio*
[2]*Department of Cell Biology, Cleveland Clinic Lerner College of Medicine, Cleveland, Ohio*
[3]*Department of Neurosurgery, Cleveland Clinic Lerner College of Medicine, Cleveland, Ohio*
[4]*Department of Molecular Medicine, Cleveland Clinic Lerner College of Medicine, Cleveland, Ohio*

KEY WORDS
ion channels; cancer; cell cycle; electrical stimulation; apoptosis

ABSTRACT
Electric fields impact cellular functions by activation of ion channels or by interfering with cell membrane integrity. Ion channels can regulate cell cycle and play a role in tumorigenesis. While the cell cycle may be directly altered by ion fluxes, exposure to direct electric current of sufficient intensity may decrease tumor burden by generating chemical products, including cytotoxic molecules or heat. We report that in the absence of thermal influences, low-frequency, low-intensity, alternating current (AC) directly affects cell proliferation without a significant deleterious contribution to cell survival. These effects were observed in normal human cells and in brain and prostate neoplasms, but not in lung cancer. The effects of AC stimulation required a permissive role for GIRK2 (or $K_{IR}3.2$) potassium channels and were mimicked by raising extracellular potassium concentrations. Cell death could be achieved at higher AC frequencies (>75 Hz) or intensities (>8.5 μA); at lower frequencies/intensities, AC stimulation did not cause apoptotic cellular changes. Our findings implicate a role for transmembrane potassium fluxes via inward rectifier channels in the regulation of cell cycle. Brain stimulators currently used for the treatment of neurological disorders may thus also be used for the treatment of brain (or other) tumors. © 2005 Wiley-Liss, Inc.

INTRODUCTION

Despite improved pharmacotherapy protocols, modern imaging and advanced surgical techniques, the prognosis for a variety of malignant cancers is still bleak. It is thus not surprising that a number of approaches have been deployed to arrest cell cycle in neoplastic cells while sparing surrounding and presumably normal cells. Although therapeutic targeting of tumor-associated mutations may be effective in tumor management, most tumor mutations arise as later stage epiphenomena of tissue disorganization and their involvement with tumor initiation, promotion, or progression has not been established conclusively (Sonnenschein and Soto, 2000; Seyfried, 2001; Mukherjee et al., 2002; Seyfried et al., 2003). Clearly, alternative therapies are needed that can better manage brain tumors while permitting a decent quality of life.

Increasing the temperature of the tumor to a level at which cancerous cells are destroyed can be used to destroy malignant tumors but is potentially noxious for the surrounding tissue. One method used for this purpose is to focus a beam of microwave energy of the type generated in a microwave oven onto the tumor. In an electrochemical procedure (Nilsson et al., 2000; Nordenstrom et al., 1994), electrodes are implanted in or around the malignant tumor to be treated. The treatment lasts several hours during one or more sessions and can be used either alone or in conjunction with other therapy, such as chemotherapy or radiation therapy. Applied across these electrodes is a low DC voltage usually having a magnitude of <10 V, causing a current to flow between the electrodes through the tumor. As a result of an electrochemical reaction, chemical products are yielded, which include cytotoxic agents that act to destroy the tumor. In particular, Na^{+}-K^{+} were altered and Cl^{-} was electrochemically segregated, yielding to a pH shift sufficient to destroy surrounding tissue (Nilsson et al., 1994).

Recently, low-intensity, kilohertz (kHz) frequency applied to replicating cells was shown to hamper cell division (Kirson et al., 2004). Experimental evidence suggested that electrical fields may interfere with cytoskeletal mechanisms responsible for the formation of mitotic spindles.

Arrest of spinal cord astrocyte growth at defined stages of the cell cycle leads to significant changes in the expression of voltage-activated Na^{+} and K^{+} currents (MacFarlane and Sontheimer, 2000). Furthermore, recent studies have shown that in quiescent glia, inhibition of inward rectifier potassium channels (K_{IR}) in-

Grant number: HL51614; Grant number: NS43284; Grant number: NS38195; Grant number: NS46513. Grants are from NIH.

*Correspondence to: Damir Janigro, Department of Neurological Surgery, NB20, The Cleveland Clinic Foundation, 9500 Euclid Ave., Cleveland, OH 44195. E-mail: janigrd@ccf.org

Received 21 September 2004; Accepted 7 January 2005

DOI 10.1002/glia.20188

Published online 18 March 2005 in Wiley InterScience (www.interscience.wiley.com).

© 2005 Wiley-Liss, Inc.

creases cell proliferation, suggesting that down regulation of K_{IR} promotes cell cycle progression through the G1/S checkpoint, while premature expression or overexpression of K_{IR} occurs when this proliferation is arrested in G1/G0 (MacFarlane and Sontheimer, 2000). This finding suggests a possible interaction between cell cycle and K_{IR} channel activity. For example, in *weaver* mice, it is now clear that a mutation in the gene coding for G-protein-activated inwardly rectifying potassium channel GIRK2 is responsible for apoptosis in the external germinal layer of the cerebellum and a nonapoptotic death of midbrain dopaminergic neurons (Patil et al., 1995; Migheli et al., 1999).

We describe a novel electrical approach for the treatment of neoplasms or other hyperplastic disorders. In this study, we tested the efficacy of very low-frequency (Hz) AC current in altering cell cycle of normal and neoplastic cells. Our results suggest a potential clinical application of electrical stimulation to reduce cell proliferation.

MATERIALS AND METHODS

Cell Isolation and Culture

Epileptic astrocyte cultures are established from human cerebral cortical tissue of patients (n = 6) undergoing temporal lobectomies to relieve medically intractable seizures. Brain resections are collected in an ice-cold solution mimicking artificial cerebrospinal fluid (ACSF) composition bubbled with 5% CO_2/95% O_2. The ACSF solution consists of (in mM): 120 NaCl, 3.1 KCl, 3 $MgCl_2$, 1 $CaCl_2$, 1.25 KH_2PO_4, 26 $NaHCO_3$, 10 dextrose. Briefly, tissue is homogenized after gentle trituration and incubation in phosphate-buffered saline (PBS) containing trypsin (0.2%)/DNase (1 mg/ml, Sigma-Aldrich, St. Louis, MO) for 20 min at 37°C. After centrifugation (15*g* for 5 min) and filtration through a 70-μm nylon sieve, the supernatant is collected, filtered through a 70-μm mesh strainer, and centrifuged again at ≈400*g* for 10 min. Cells are then seeded in an appropriate poly-D-lysine-coated flask or in 6- or 24-multiwell plates. After reaching confluence, the cultures are agitated overnight at 37°C. Cytosine arabinoside and L-leucine methyl ester (Sigma-Aldrich) are added to obtain a highly purified astrocytic population (Meyer et al., 1991). Before utilization, immunological characterization is performed with rabbit polyclonal antibodies that recognize the glial marker glial fibrillary acidic protein (GFAP; Dako, Carpenteria, CA). Rat glioma C6 (Cat. no. CCL-107), prostate tumor (PC-3, Cat. no. CRL-1435), and lung tumor (H1299 Cat. no. CRL-5803) cell lines were purchased from American Type Cell Culture (ATCC). Normal human astrocytes were purchased from TCS Cellworks (Cat. no. sc-1810). Cells were grown in Dulbecco's modified essential medium (DMEM-F12) supplemented with 2 mM glutamine, 10% fetal bovine serum (FBS), 100 U of penicillin G sodium per ml, and 100 μg of streptomycin sulfate per ml. Cells were maintained at 37°C in a humidified atmosphere consisting of 5% CO_2 and 95% air.

In Vitro Modulation of Cell Growth by Electrical Stimulation

Cells were grown into 24-well plates. The seeding density was $1 \cdot 10^4/cm^2$. The plate is engineered to accommodate a pair of stainless steel electrodes per well, connected to a waveform generator. The stimulation starts 1 day after the initial cell seeding and keeps going 24 h/day for 5 days consecutively. The peak surface current density was calculated by an automated 2D finite-elements approximation, assuming a purely resistive system. Each well was divided into 360 sub-elements, and Kirchhoff's laws applied according to: $\sum I = 0$ (for every node) and $\sum V = 0$ (for every closed loop). The resulting current, divided by the element area, was used to calculate surface current density. Cellular growth was monitored every day by inspection with phase-contrast microscopy. Pictures from each well were taken using a 35-mm camera mounted on the microscope unit, and interfaced to a PC using Qcapture software (Nonlinear USA Inc., Durham, NC). The images were analyzed and the cells counted by Phoretix 2D Image Analysis Software.

In Vitro Modulation of Cell Growth by Extracellular Potassium

Cells were seeded into pre-coated 24-well plates as described above. Cells were then exposed to scalar concentration of K^+ (from a 4 mM basal $[K]_{OUT}$) by adding KCl to the growth media in order to achieve experimental $[K]_{OUT}$ values (5, 8, 12, and 48 mM). Parallel cultures were exposed to corresponding concentration of NaCl to evaluate whether manipulations of osmolarity alone were effective. Cellular growth was monitored daily by inspection with phase-contrast microscopy as described above.

Adenylate Kinase Measurement

Detection of cytotoxicity and cytolysis was assessed by measurement of the release of adenylate kinase (AK). Media samples were taken after 5 days of stimulation protocol, while cell proliferation was assessed by phase-contrast microscopy. The measurements were performed by the use of the ToxiLight™ HS kit (Cambrex Bio Science, Rockland, ME). The assays were conducted at ambient temperature (18–22°C) following the procedure described by the manufacturer (Kohler et al., 1999).

Monitoring of Thermal Effects

To rule out the possibility that electrical stimulation might induce temperature-dependent changes, culture media were exposed to the same patterns of electrical stimulation used for our experiments (see above). A 24-well plate filled with tissue culture media (pre-heated to 33°C) was placed in the incubator at 37°C, and temperature measured at a 30-min interval over a 12-h period with a thermistor probe connected to a telethermometer (model 43 TD, Yellow Springs Instrument Company, Yellow Springs, OH).

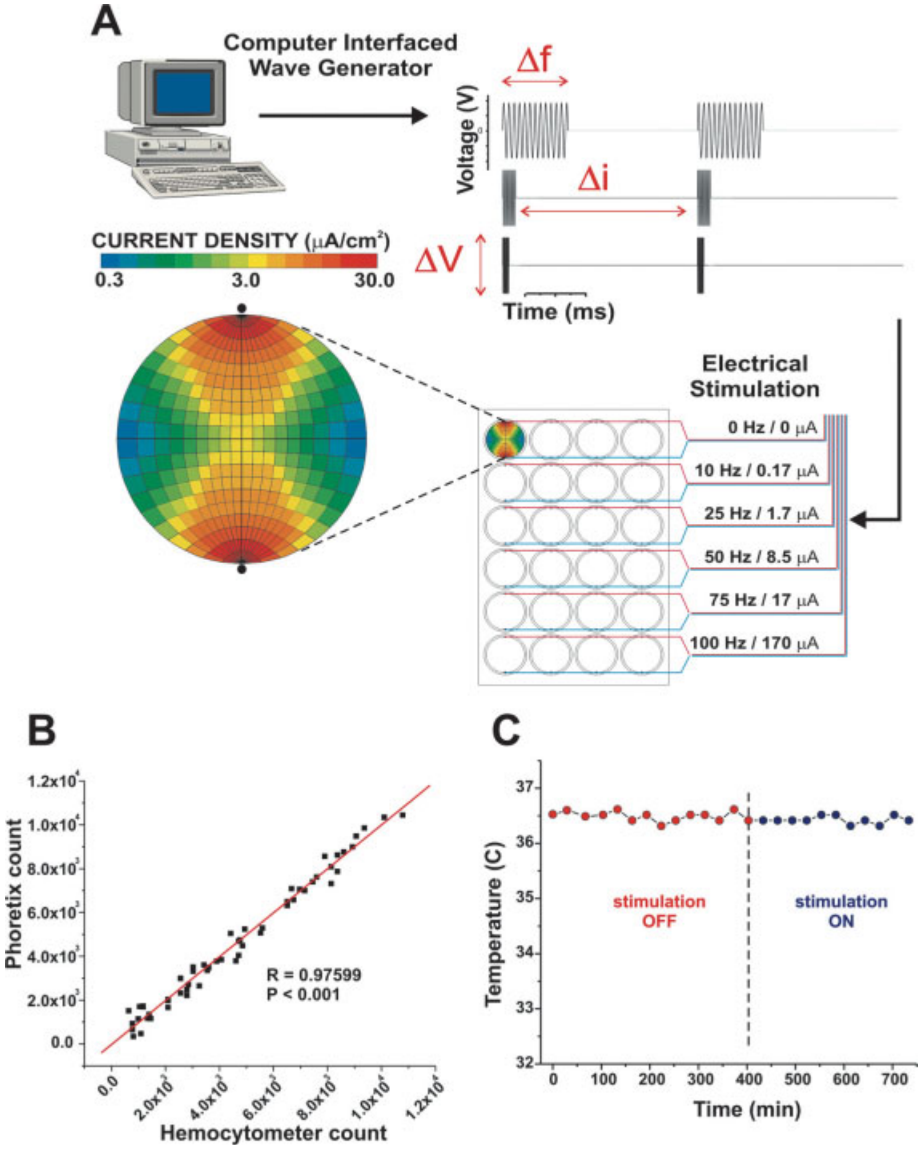

Fig. 1. Experimental setup. **A:** Schematics of the experimental set up. Current densities obtained when stimulating at 7.5 μA, 50 Hz are also shown. The pattern of stimulation and the variables that were manipulated (frequency and intensity) are also shown. **B:** Cells were counted by using a computer image analysis technique, or by a more conventional hemocytometer method. The correlation between the two methods is statistically significant between the two methods (R = 0.97599). **C:** Effect of AC stimulation on culture media temperature. Prolonged exposure to electrical stimulation (blue dots), did not result in any significant change in temperature compared with the unstimulated condition (red dots).

BrdU Labeling and Cell Proliferation

Cells were grown as previously described by others for 4–6 days under either stimulated or nonstimulated conditions. Cells were incubated with BrdU (BD Biosciences Pharmingen^{TM} in situ Detection Kit) for 3 h at a final concentration of 10 μM. The cell culture density was in compliance with the manufacturer specifications. Cells from the same population that are not BrdU-labeled were the negative cell staining control. Cell proliferation was measured in the same cultures.

Western Blot Analysis

Cell extracts from stimulation experiments were scraped and dissolved in RIPA buffer containing protease inhibitors (0.17 mg/ml PMSF, 2 μg/ml leupeptin, and 0.7 μg/ml aprotinin). Prior to electrophoresis, protein extracts were denatured by heating at 100°C for 5 min in a running buffer solution containing RIPA, β-mercaptoethanol, and bromophenol blue tracking dye. 15 μg protein were loaded in each lane. Duplicate acrylamide gels (12%, precast gels; Bio-Rad, Hercules, CA) were run for 2.5–3 h at constant voltage (80 V) until the bromophenol blue tracking dye migrated to the bottom edge of the gels. Proteins were then transferred onto a blot of PVDF using constant current (40 mA) overnight at 4°C. Proteins were probed overnight at 4°C with primary Kir3.2 rabbit anti-human antibody (1:500; Upstate Biotechnology, Lake Placid, NY). Blots were washed and treated with Goat Anti-Rabbit IgG horseradish peroxidase (HRP)-conjugated secondary antibody (1:5,000; Dako). To ensure that the same amount of total protein was electroblotted, PVDF membranes were incubated for 20 min at 37°C in a "stripping buffer" (Restore Western Blot Stripping Buffer; Pierce, Rockford, IL). Nonspecific binding blocking was performed as described above; membranes were reprobed with monoclonal anti-β-Actin antibody (1:10,000; Sigma-Aldrich).

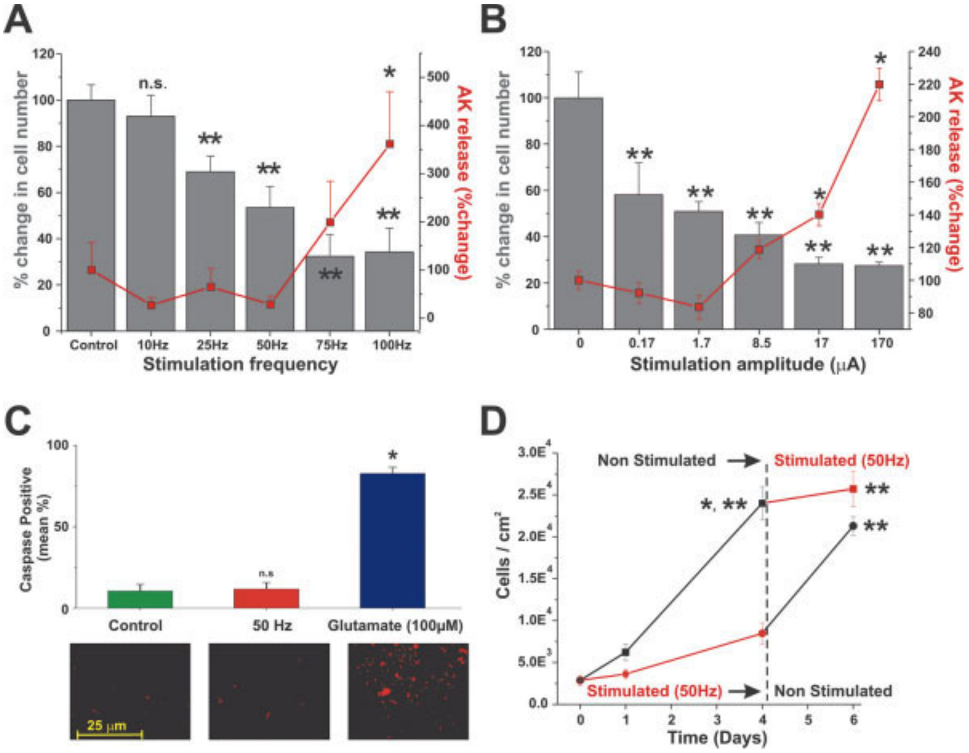

Fig. 2. Dissociation of cytotoxic and cytostatic effects of AC stimulation. **A:** Release of adenylate kinase (red squares) and cell proliferation (graph bars) in response to AC electrical stimulation depends on stimulation frequency. AK was measured in culture media exposed to ES for 5 days. Note that frequencies of >75 Hz have a significant impact on cell viability, as indicated by the increased release of AK. **B:** Release of adenylate kinase (red squares) and cell proliferation (graph bars) in response to AC electrical stimulation depend on stimulus intensity. **C:** Caspase-3 assays were performed on stimulated and naive cells. As a positive control, cells were exposed to glutamate at a concentration of 100 μM for 1 h to induce apoptosis. No significant differences were revealed in cell death between stimulated and nonstimulated cultures ($P = 0.353$, $n = 3$, ±SEM). **D:** The stimulation pattern was sometimes reversed to demonstrate that stimulated cells promptly reentered cell cycle, while onset of stimulation on nonstimulated cells arrested proliferation without significantly reducing proliferation. The data represent a mean of 5 experiments. Data significantly different ($P < 0.05$) from stimulated cells (*) and from pre-stimulation (**) values.

Caspase-3 Immunohistochemical Detection

Cells were grown on glass coverslips. The assays were conducted following the procedure described by the manufacturer (Anti-ACTIVE® Caspase-3 pAb; Promega, Madison, WI). As positive control, glutamate-induced apoptosis was assessed in human astrocytes. Cells were exposed to glutamate at a concentration of 100 μM for 1 h. The media were renewed, and the cells were allowed to remain in culture for 24 h prior to Caspase-3 immunohistochemistry with a Texas Red-conjugated secondary antibody (Jackson ImmunoResearch, West Grove, PA). Three random cell counts were taken from each coverslip in every condition. Both total cell number and caspase-3-positive cells were quantified. Statistical analysis was performed with Sigma Stat 2.0 software, Jandel Scientific, San Rafael, CA).

Anti-GIRK2, NT (Kir 3.2) Immunohistochemical Detection

To investigate the expression of GIRK2 protein in human prostate or lung cancer cell lines, cells were grown on coverslips and exposed to stimulation or nonstimulation for 3 days. Cells were then fixed in formalin, and coverslips were incubated for 1 h at RT with primary antibody: Anti-GIRK2, NT (Kir3.2) rabbit polyclonal IgG (1:100; Upstate Biotechnology). Coverslips were then rinsed 5× in PBS and incubated for 1 h at RT in the dark with secondary antibody fluorescein isothiocyanate (FITC)-conjugated Affini-Pure Donkey Anti-Rabbit IgG (1:200; Jackson ImmunoResearch). Coverslips were rinsed a final time with 1× PBS and mounted on glass slides, using Vectashield mounting medium with DAPI (Vector, Burlingame, CA) and analyzed by fluorescent microscopy (Leica Leitz DM-RXE).

RESULTS

Cell cultures were initially grown in 24-well Petri dishes equipped with an array of stainless steel electrodes connected to a computer via an I/O board (Fig. 1A). Each well was modified to accommodate two stainless steel electrodes (series resistance ~2 Ω. After plating on either plastic or glass inserts, cells were exposed to a computer-controlled electrical stimulation protocol. Cells were exposed to 50-Hz AC (current intensity ≃7.5 μA 32 cycles/pulse, 10-s interval between each pulse) for

3–5 days. Cell number was monitored daily with phase-contrast microscopy. This technique was validated in parallel hemocytometry methods (Fig. 1B). Viability was assessed by determination of Caspase-3 immunoreactivity. The stimulation parameters used in our experiment did not induce temperature-dependent effects (Fig. 1C).

Cells exposed to stimulation at 10 Hz for 2–5 days grew at a rate similar to that of unstimulated glia ($P = 0.2$, Fig. 2A). In contrast, stimulation at 25–100 Hz caused a pronounced decrease in cell density as early as three days after stimulation. The effects persisted and amplified with prolonged exposure to electric pulses. We hypothesized that a reduction in cell proliferation rather than cell death was responsible for the decreased cell density occurring in stimulated wells. Stimulation of $\leq$ 50 Hz decreased cell number through a direct effect on cell cycle, and not cell death, as evidenced by the assessment of adenylate kinase (AK) release (Single et al., 1998), confirmed by caspase-3 immunoassay, as shown in Figure 2A–C. Incorporation of BrdU in stimulated cells is also greatly diminished in comparison to the controls, further demonstrating a direct effect of electrical stimulation on cell cycle (Fig. 3A). Furthermore, a direct effect on cell cycle, rather than toxicity, was implicated as the underlying mechanism, due to the reversible nature of this phenomenon. Figure 2D shows that cell proliferation was restored after cessation of AC stimulation. At frequencies of >50 Hz, the effects on cell proliferation overlapped significantly with a negative effect on cell survival, as demonstrated by the sharp increase in AK release. Next, we examined the dependency of cell viability/proliferation on stimulation intensity and found that applying current intensities higher than 8.5 μA causes cellular damage as revealed by a statistical significant increase of AK release (Fig. 2B). Thus, excessive frequency/intensity AC cell stimulation caused cell toxic effects similar to electrotherapy. AC delivered at low-frequency/intensity resulted in decreased cell proliferation without any significant deleterious effects on viability.

To investigate whether electric current can affect proliferation in cells other than glia, we tested the effect of stimulation at 50 Hz/7.5 μA on C6 rat glioma, a human prostate cancer cell line (PC-3), and a lung cancer cell line (H1299). Stimulation affected glioma and PC-3 proliferation but did not alter the rapid expansion of lung cancer cells, suggesting that its effects were downstream from common nuclear events involved in cell cycle regulation (Fig. 3B).

Abnormal electrical activity affects the expression of G-protein-coupled inward rectifying channels (Pei et al., 1999). We tested expression of $K_{IR}3.2$ (or GIRK2, KCNJ6) in glial, lung cancer and prostate cancer cells prior to and following electrical stimulation. Lung cancer cells expressed low basal levels of GIRK2, and in these cells expression was not affected by electrical stimulation. In contrast, prostate cancer cells and human astrocytes expressed higher basal levels of GIRK2, and its expression was drastically increased after 5 days of stimulation. This was assessed by immunohistochemical

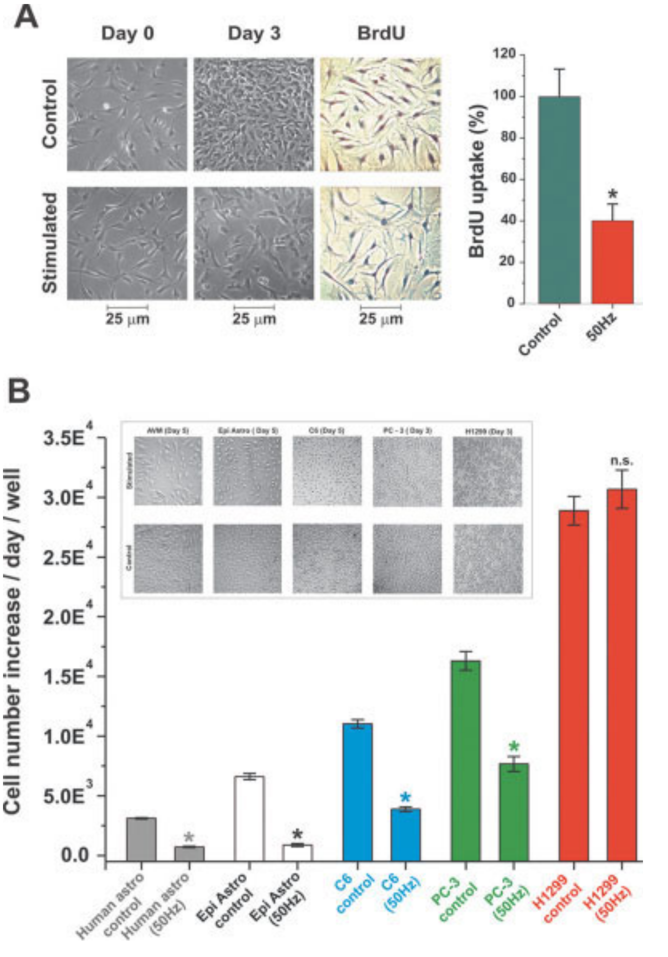

Fig. 3. Effect of AC stimulation on cell proliferation. **A:** Incorporation of BrdU was significantly reduced in cells exposed to electrical stimulation in comparison to control cells, consistently with an anti-proliferative effect of AC stimulation. **B:** Normal and epileptic human astrocytes, C6, and PC-3 cell lines responded to electrical stimulation, while the H1299 lung tumor cell line did not (see text for details). Each experiment was performed in triplicate; $^*P < 0.05$; n.s., not significant. Error bars reflect SEM.

detection on cultured cells and confirmed by Western blot (Fig. 4). These results suggest that expression of GIRK2 is causally related to the effects of stimulation.

We tested whether changes in membrane potential may affect proliferation. This was achieved by exposing unstimulated astrocytes to increasing concentrations of KCl (Fig. 5A). Osmolarity was preserved by removing an equivalent amount of NaCl. The concentration-dependent inhibitory effects of extracellular potassium on proliferation were similar to electrical stimulation, since the effect of elevated $[K]_{OUT}$ was readily reversible and not due to cell death. Manipulations of extracellular sodium levels alone from 5 mM to 48 mM were ineffective (Fig. 5A, inside panel). These results suggest that depolarization via potassium influx may lead to decreased proliferation of human astrocytes.

To test the hypothesis that permeation of potassium ions was required to observe decreased proliferation by either increased expression of K_{IR} or elevated $[K]_{OUT}$, we

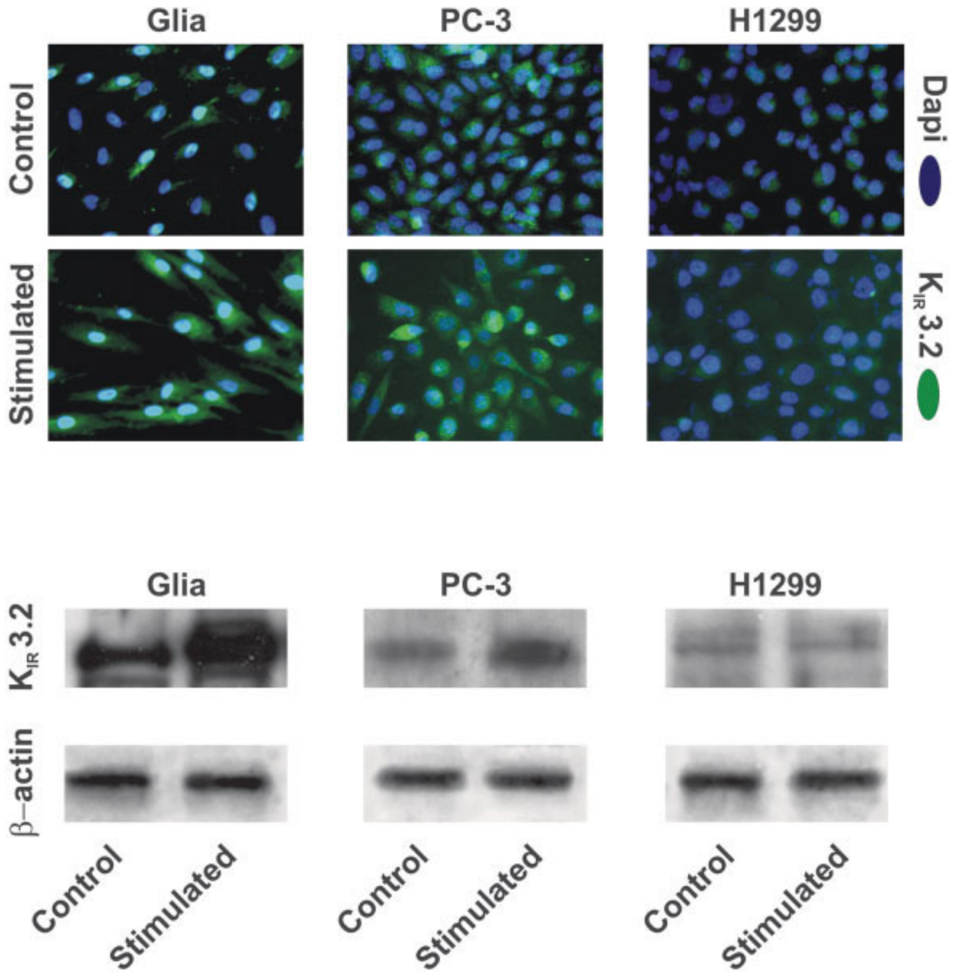

Fig. 4. Overexpression of the inward rectifier potassium channel Kir3.2 in cells respondent to AC stimulation. Expression of K_{IR}3.2 in human astrocytes, H1299 and PC-13 cells was assessed by immunocytochemical analysis. AC stimulation increased basal levels of expression of K_{IR}3.2 in PC-3 and astrocytes, but not in H1299 cells. The data were confirmed by Western blot.

exposed lung cancer cells (which do not respond to electrical stimulation), prostate cancer cells and human astrocytes (whose proliferation is inhibited by electrical stimulation) to the K_{IR} blocker, cesium (0.1 mM) (Ransom and Sontheimer, 1995; D'Ambrosio et al., 1999). As expected, lung cancer cells did not respond to stimulation or cesium, while cesium abolished the anti-proliferative effects of electrical stimulation in astrocytes and PC-3 cells (Fig. 5B). Similar results were obtained using 0.1 mM barium, another K_{IR} blocker.

DISCUSSION

The main finding presented is that very low-intensity AC current can dramatically reduce cell proliferation by a mechanism implicating specific potassium channels. Previous work by other investigators demonstrated a cytotoxic action caused by direct current (Humphrey and Seall, 1959; Holandino et al., 2001) or an antiproliferative effect at kHz frequencies (Kirson et al., 2004). In our experiments, cellular damage occurred only at elevated frequencies and intensities. This was assessed by two independent methods, involving the monitoring of caspase-3 and AK activities. In addition, the effect of AC electrical stimulation on cell proliferation proved to be fully reversible (Fig. 2D). The data presented also implicate the selective expression and activity of an inward rectifier potassium channel, GIRK2 (or K_{IR}3.2) whose role in the cell cycle and expression in human glia was previously unrecognized. Notably, a previous report linked electroconvulsive therapy presumably to neuronal expression of the same ion channel family (Pei et al., 1999) suggesting that anomalous patterns of cellular excitation may trigger GIRK2 expression.

A direct, causal role for K_{IR} in electrical stimulation was supported by (1) the lack of anti-proliferative actions in H1299 cells where AC stimulation fail to induce overexpression of K_{IR}; (2) the fact that enhancement of inward potassium (but not sodium) fluxes mimicked the effects of K_{IR} (over)expression, and (3) the obliterating effects of the voltage-dependent blocker cesium at concentrations that are specific for inwardly rectifying potassium channels (Ransom and Sontheimer, 1995). In addition, K_{IR}3.2 levels were increased (2-fold) in cells where the stimulation caused decreased proliferation. K_{IR}3.2 is thought to associate with K_{IR}3.1 to form channel heteromers in heart tissue. In brain, K_{IR}3.2 homomers may exist, although they may contain combinations of the three splice variants of K_{IR}3.2 that have been

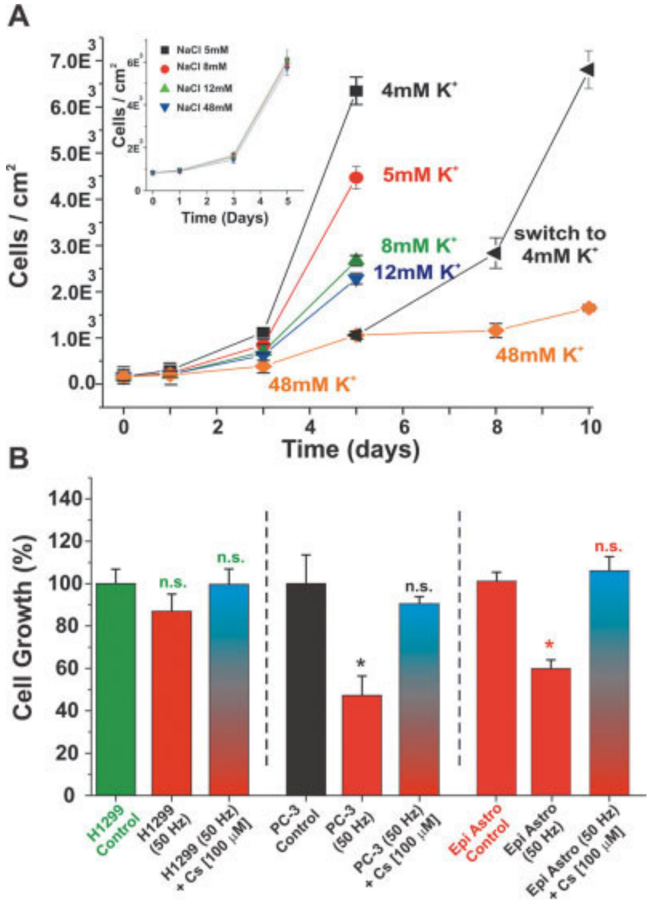

Fig. 5. Inward rectifier potassium channels are involved in cell proliferation and stimulation-induced cell cycle decrease. **A:** Concentration-dependent inhibitory effects of extracellular potassium on proliferation. Note the similarity with the effect of the electrical stimulation. Inhibition of cell proliferation at elevated $[K]_{OUT}$ was readily reversible and not due to cell death or change in the osmolarity since manipulations of extracellular sodium were ineffective (inside panel). **B:** Correlation between sensitivity to extracellular cesium, expression of $K_{IR}3.2$ (GIRK2) and the anti-proliferative effectiveness of electrical stimulation. Cells were exposed to either control media ± stimulation, or cesium (100 μM) ± stimulation (n = 6; bars reflect SEM; $^*P < 0.05$; n.s., not significant compared with unstimulated).

identified (Reimann and Ashcroft, 1999). Because GIRKs form complexes, it is difficult at this point to assess whether $K_{IR}3.2$ alone is relevant for the observed effects.

How AC may influence the cell cycle remains unknown. It is well recognized that low mitotic activity is associated with electrical excitability, since excitable cells such as neurons or heart muscle rarely give rise to tumors (Jemal et al., 2004). Similarly, while brain tumors are most devastating, their frequency in the general population is exceedingly low (Jemal et al., 2004). Clinical evidence also shows that long-term chronic epilepsy preceding formation of gliomas decreases mortality significantly (Luyken et al., 2003), perhaps suggesting that synchronous, oscillatory, and periodic abnormal electrical activity is not permissive for cell proliferation.

A recent report has shown that AC delivered at much higher frequencies (100–300 kHz) may also affect cell cycle (Kirson et al., 2004). Our results are substantially different inasmuch we exploited frequencies that are close to those measured during normal neuronal function (Buzsaki et al., 1983) and implicate a mechanism that may be of physiological relevance. One may speculate that the low incidence of tumor or other neoplastic disorders in excitable tissue may be in part due to electrical field potentials and potassium fluxes present in these cells. Similarly, cells that dwell in proximity of electrical activity (e.g., glia) give rise to tumors at a much lower rate than tissue that is exposed to electrical silence.

One of the limitations of the use of AC to treat tumors is the transient nature of the effect on cell proliferation. This may seriously limit the usefulness of this approach. Our preliminary results show that AC current stimulation may enhance chemotherapeutic efficiency, as determined by quantifying doxorubicin effects of stimulated vs. naive cells (data not shown). Thus, stimulation of gliomas achieves a synergistic effect by decreasing proliferation and decreasing intrinsic drug resistance.

In conclusion, we have shown that AC may profoundly influence cell proliferation by a mechanism that involves inward rectifying K channels. Since proliferation of prostate cancer and glioma cells was inhibited by AC, we suggest that electrical stimulation by either brain stimulators (Benabid, 2003) or peripheral electrodes (Miles et al., 1974) may have potential application in the treatment of tumor growth by reducing neoplastic cell division without significant damage to the surrounding healthy tissue.

ACKNOWLEDGMENTS

The authors thank Jordan S. Thomas for the assistance provided during the experimental work. This work was supported by HL51614, NS43284, NS38195, and NS46513 (to D.J.). All grants from NIH.

REFERENCES

Benabid AL. 2003. Deep brain stimulation for Parkinson's disease. Curr Opin Neurobiol 13:696–706.

Buzsaki G, Leung LW, Vanderwolf CH. 1983. Cellular bases of hippocampal EEG in the behaving rat. Brain Res 287:139–171.

D'Ambrosio R, Maris DO, Grady MS, Winn HR, Janigro D. 1999. Impaired K homeostasis and altered electrophysiological properties of post-traumatic hippocampal glia. J Neurosci 19:8152–8162.

Holandino C, Veiga VF, Rodrigues ML, Morales MM, Capella MA, Alviano CS. 2001. Direct current decreases cell viability but not P-glycoprotein expression and function in human multidrug resistant leukemic cells. Bioelectromagnetics 22:470–478.

Humphrey CE, Seall EH. 1959. Biophysical approach toward tumor regression in mice. Science 130:388–390.

Jemal A, Tiwari RC, Murray T, Ghafoor A, Samuels A, Ward E, Feuer EJ, Thun MJ. 2004. Cancer statistics, 2004. CA Cancer J Clin 54:8–29.

Kirson ED, Gurvich Z, Schneiderman R, Dekel E, Itzhaki A, Wasserman Y, Schatzberger R, Palti Y. 2004. Disruption of cancer cell replication by alternating electric fields. Cancer Res 64:3288–3295.

Kohler C, Gahm A, Noma T, Nakazawa A, Orrenius S, Zhivotovsky B. 1999. Release of adenylate kinase 2 from the mitochondrial intermembrane space during apoptosis. FEBS Lett 447:10–12.

Luyken C, Blumcke I, Fimmers R, Urbach H, Elger CE, Wiestler OD, Schramm J. 2003. The spectrum of long-term epilepsy-associated tumors: long-term seizure and tumor outcome and neurosurgical aspects. Epilepsia 44:822–830.

MacFarlane SN, Sontheimer H. 2000. Changes in ion channel expression accompany cell cycle progression of spinal cord astrocytes. Glia 30:39–48.

Meyer J, Tauh J, Galla HG. 1991. The susceptibility of cerebral endothelial cells to astroglial induction of blood-brain barrier enzymes depends on their proliferative state. J Neurochem 57:1971–1977.

Migheli A, Piva R, Casolino S, Atzori C, Dlouhy SR, Ghetti B. 1999. A cell cycle alteration precedes apoptosis of granule cell precursors in the weaver mouse cerebellum. Am J Pathol 155:365–373.

Miles J, Lipton S, Hayward M, Bowsher D, Mumford J, Molony V. 1974. Pain relief by implanted electrical stimulators. Lancet 1:777–779.

Mukherjee P, El Abbadi MM, Kasperzyk JL, Ranes MK, Seyfried TN. 2002. Dietary restriction reduces angiogenesis and growth in an orthotopic mouse brain tumour model. Br J Cancer 86:1615–1621.

Nilsson E, von Euler H, Berendson J, Thorne A, Wersall P, Naslund I, Lagerstedt AS, Narfstrom K, Olsson JM. 2000. Electrochemical treatment of tumours. Bioelectrochemistry 51:1–11.

Nordenstrom BE. 1994. Electrostatic field interference with cellular and tissue function, leading to dissolution of metastases that enhances the effect of chemotherapy. Eur J Surg (suppl) 1994; 574: 121–135.

Patil N, Cox DR, Bhat D, Faham M, Myers RM, Peterson AS. 1995. A potassium channel mutation in weaver mice implicates membrane excitability in granule cell differentiation. Nat Genet 11:126–129.

Pei Q, Lewis L, Grahame-Smith DG, Zetterstrom TS. 1999. Alteration in expression of G-protein-activated inward rectifier K^+-channel subunits GIRK1 and GIRK2 in the rat brain following electroconvulsive shock. Neuroscience 90:621–627.

Ransom CB, Sontheimer H. 1995. Biophysical and pharmacological characterization of inwardly rectifying potassium currents in rat spinal cord astrocytes. J Neurophysiol 73:333–346.

Reimann F, Ashcroft FM. 1999. Inwardly rectifying potassium channels. Curr Opin Cell Biol 11:503–508.

Seyfried TN. 2001. Perspectives on brain tumor formation involving macrophages, glia, and neural stem cells. Perspect Biol Med 44:263–282.

Seyfried TN, Sanderson TM, El Abbadi MM, McGowan R, Mukherjee P. 2003. Role of glucose and ketone bodies in the metabolic control of experimental brain cancer. Br J Cancer 89:1375–1382.

Single B, Leist M, Nicotera P. 1998. Simultaneous release of adenylate kinase and cytochrome c in cell death. Cell Death Differ 5:1001–1003.

Sonnenschein C, Soto AM. 2000. Somatic mutation theory of carcinogenesis: why it should be dropped and replaced. Mol Carcinog 29:205–211.

Tsuruo T, Naito M, Tomida A, Fujita N, Mashima T, Sakamoto H, Haga N. 2003. Molecular targeting therapy of cancer: drug resistance, apoptosis and survival signal. Cancer Sci 94:15–21.
